# Supplementary material for: Histone H2AK119 Mono-Ubiquitination Is Essential for Polycomb-Mediated Transcriptional Repression
Source: Mol Cell. 2020 Feb 20;77(4):840–856.e5. doi: 10.1016/j.molcel.2019.11.021 (PMC7033561; doi:10.1016/j.molcel.2019.11.021)
Supplement: Document S1. Figures S1–S2 [file mmc1.pdf]

Molecular Cell, Volume *77*

Supplemental Information

Histone H2AK119 Mono-Ubiquitination Is Essential for Polycomb-Mediated Transcriptional Repression

Simone Tamburri, Elisa Lavarone, Daniel Fernández-Pérez, Eric Conway, Marika Zanotti, Daria Manganaro, and Diego Pasini

Figure S1

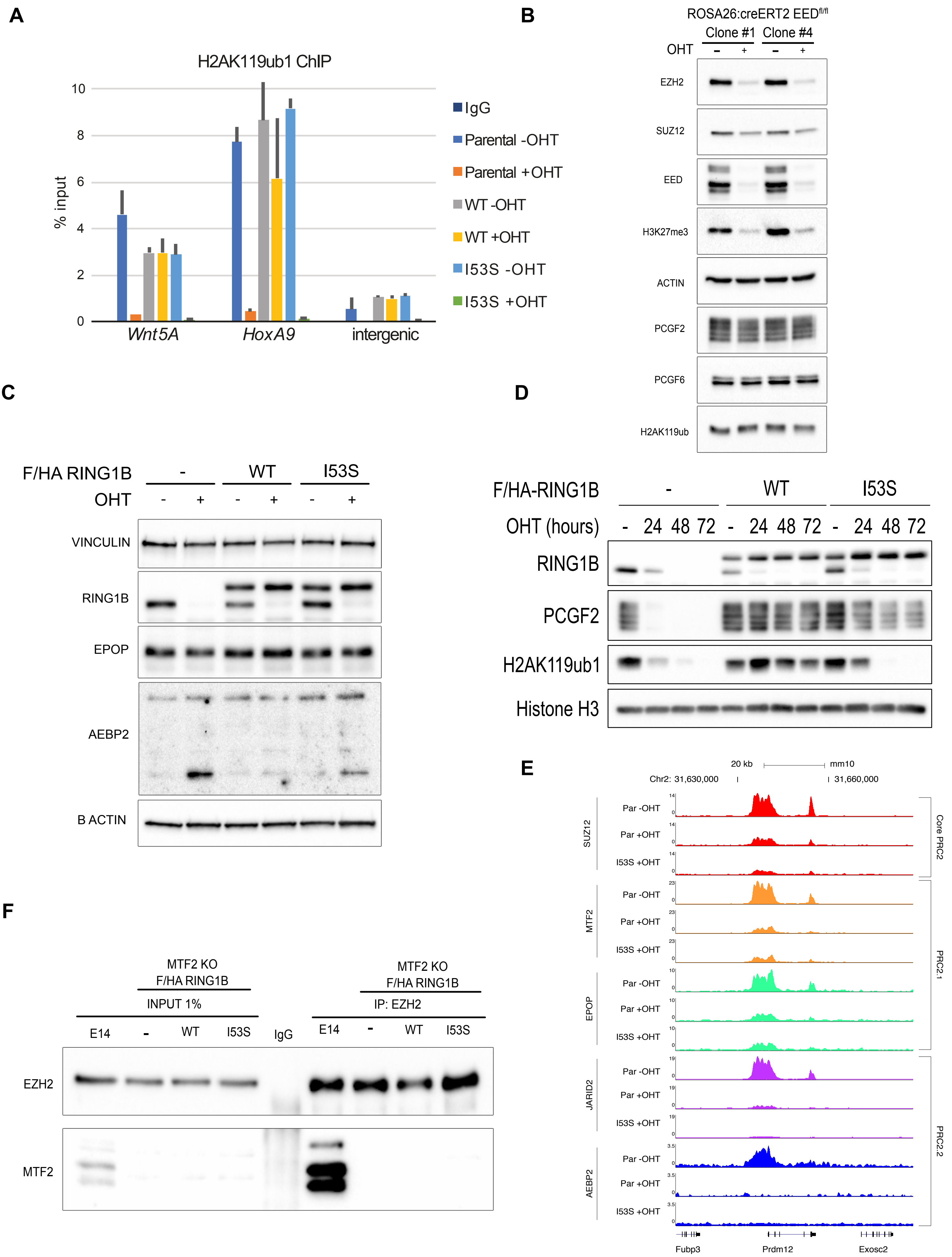
A
H2AK119ub1 ChIP
% input
10
8
6
4
2
0
Wnt5A
HoxA9
intergenic
IgG
Parental -OHT
Parental +OHT
WT -OHT
WT +OHT
I53S -OHT
I53S +OHT
B
ROSA26:creERT2 EED fl/fl
Clone #1
Clone #4
OHT
EZH2
SUZ12
EED
H3K27me3
ACTIN
PCGF2
PCGF6
H2AK119ub
C
F/HA RING1B
-
WT
I53S
OHT
VINCULIN
RING1B
EPOP
AEBP2
B ACTIN
D
F/HA-RING1B
OHT (hours)
24 48 72
RING1B
PCGF2
H2AK119ub1
Histone H3
E
20 kb
mm10
Chr2: 31,630,000
31,660,000
SUZ12
MTF2
EPOP
JARID2
AEBP2
Par -OHT
Par +OHT
I53S +OHT
Core PRC2
PRC2.1
PRC2.2
Fubp3
Prdm12
Exosc2
F
MTF2 KO
F/HA RING1B
INPUT 1%
IP: EZH2
E14
WT
I53S
IgG
EZH2
MTF2

Figure S1. Related to Figure 1 to 6.

(A) ChIP-qPCR analysis of H2AK119ub1 in the indicated cell lines at two specific Polycomb targets and one intergenic region. IgG served as a negative control. Related to Figure 1.

(B) Western blot analysis with the indicated antibodies of total protein extracts obtained from the specified cell lines upon 72 hours of treatment with OHT (+OHT) or EtOH (-OHT). ACTIN was used as a loading control. Related to Figure 2.

(C) Western blot analysis with the indicated antibodies of total protein extracts obtained from the specified cell lines upon 72 hours of treatment with OHT (+OHT) or EtOH (-OHT). ACTIN and VINCULIN were used as a loading control. Related to Figure 3.

(D) Western blot analysis with the indicated antibodies of total protein extracts obtained from the specified cell lines at the indicated time of treatment with OHT (+OHT) or EtOH (-OHT). H3 was used as a loading control. Related to Figure 4.

(E) Representative genomic snapshots of ChIP tracks of the indicated proteins at the PRDM12 gene locus. Related to Figure 4.

(F) Co-immunoprecipitation analysis of total extracts derived from parental and FLAG-HA (F/HA) -tagged RING1B WT or I53S expressing cells upon 72 hours of treatment with OHT (+OHT) or EtOH (-OHT) using EZH2 antibody crosslinked to HA beads. E14 mESCs served as positive control for MTF2 co-immunoprecipitation upon EZH2 IP. IgG-IPs in E14 mESCs cells served as a negative control. Related to Figure 5.

Figure S2

A

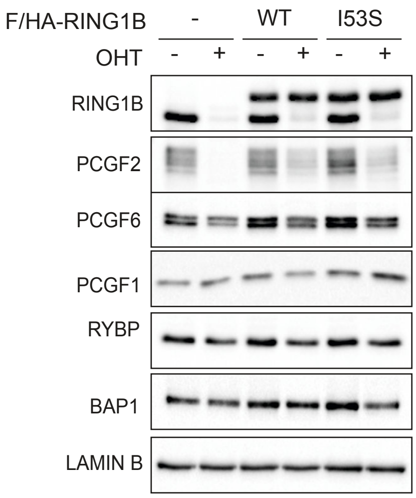

B

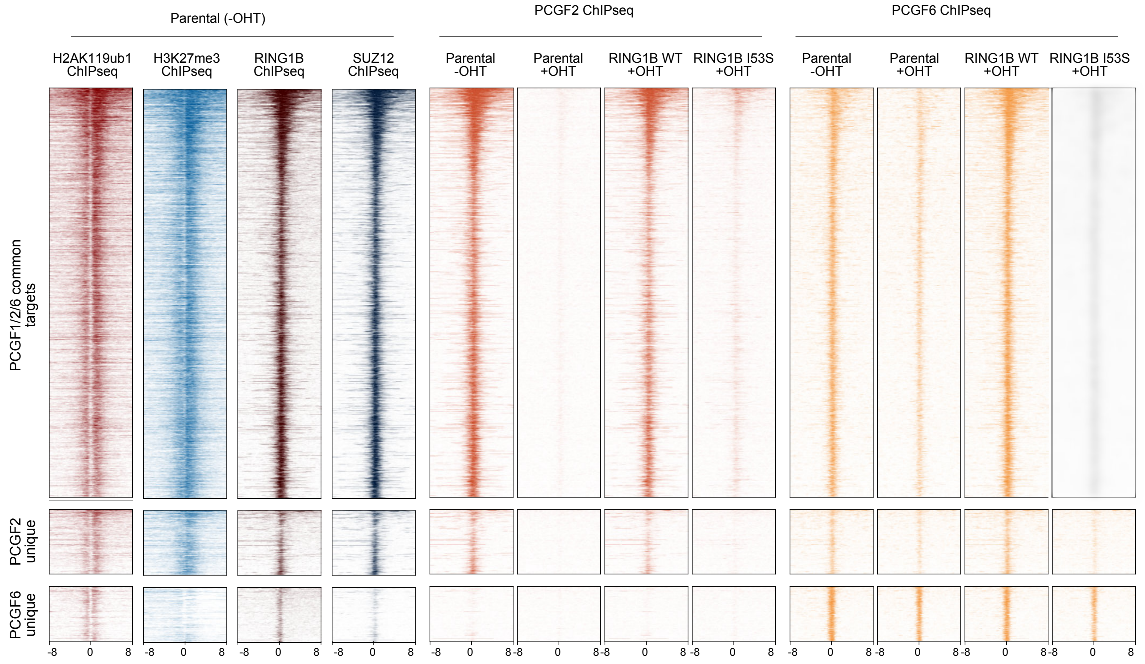

C

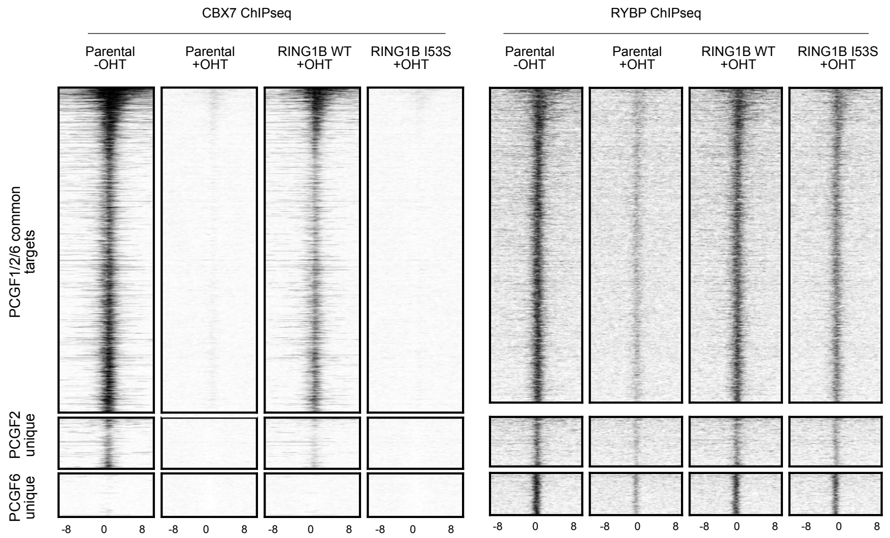

Figure S2. Related to Figure 7.

(A) Western blot analysis with the indicated antibodies of nuclear protein extracts obtained from the specified cell lines upon 72 hours of treatment with OHT (+OHT) or EtOH (-OHT). LAMIN B served as a negative control.

(B) Heatmaps representing normalized H2AK119ub1, H3K27me3, RING1B, SUZ12, PCGF2 and PCGF6 ChIP-seq intensities ± 8Kb around the TSS of PCGF target genes in the indicated cell lines.

(C) Heatmaps representing normalized CBX7 and RYBP ChIP-seq intensities ± 8Kb around the TSS of PCGF target genes in the indicated cell lines.

Table S1. Mass spectrometry analysis of RING1B WT and I53S mutant partners. Related to Figure 1

Values of the LFQ ratios of the RING1B interactors obtained by MS/MS analyses in the FLAG-HA (F/HA)-tagged RING1B WT or I53S immuno-purifications (anti-FLAG) from mESCs.

Table S2. ChIPseq targets. Related to Figure 1

List of all RING1B and H2AK119ub1 peaks (target loci) found in WT +OHT cell line sorted by RING1B and H2AK119ub1 binding, respectively, and PCGFs target genes obtained from (31029542) liftOver to mm10 and sorted by RING1B binding.

Table S3. RNAseq analysis upon loss of PRC1 enzymatic activity. Related to Figure 2

List of all RefSeq genes and their corresponding RNA-seq results for the indicated cell lines at 72 hours from EtOH (-OHT) or OHT (+OHT) treatment.
